# Supplementary material for: Longitudinal clonal dynamics of HIV-1 latent reservoirs measured by combination quadruplex polymerase chain reaction and sequencing
Source: Proc Natl Acad Sci U S A. 2022 Jan 18;119(4):e2117630119. doi: 10.1073/pnas.2117630119 (PMC8794825; doi:10.1073/pnas.2117630119)
Supplement: Supplementary File [file pnas.2117630119.sapp.pdf]

PNAS
www.pnas.org

Supplementary Information for

Longitudinal clonal dynamics of HIV-1 latent reservoirs measured by combination quadruplex polymerase chain reaction and sequencing

Alice Cho[1], Christian Gaebler[1], Thiago Olveira[1], Victor Ramos[1], Marwa Saad[1], Julio Lorenzi[1], Ana Gazumyan[1], Susan Moir[3], Marina Caskey[1], Tae-Wook Chun[3], Michel Nussenzweig[1, 2]

[1]Laboratory of Molecular Immunology, The Rockefeller University, New York, NY 10065, USA
[2]Howard Hughes Medical Institute
[3]Laboratory of Immunoregulation, National Institute of Allergy and Infectious Diseases (NIAID)

Correspondence to Michel C. Nussenzweig
Email: nussen@rockefeller.edu

This PDF file includes:

Table S1
Figure S1
Figure S2
Table S2
Figure S3

Table S1. Characteristics of study participants.

Suggested Participant ID	Gender	Race	Early or chronic at the time of ART initiation	Time after infection when ART was initiated (day)	Plasma viremia prior to ART	CD4 count prior to ART	CD4 % prior to ART	CD8 count prior to ART	CD8 % prior to ART	ART regimen
P1	M	White	Chronic	447	25,689	329	29	511	45	Efavirenz/Emtricitabine/ Tenofovir DF
P2	M	Black	Chronic	2,879	19,957	465	24	1,066	55	Tenofovir/Emtricitabine/ Atazanavir/ Ritonavir
P3	M	Unknown- Hispanic/ Latino	Chronic	417	23,033	294	25	400	34	Efavirenz/Emtricitabine/ Tenogovir
P4	M	White	Chronic	100	27,714	135	9	856	57	Tenofovir/Emtricitabine/ Atazanavir/ Ritonavir
P5	M	White	Chronic	414	8,690	472	34	375	27	Rilpivirine/Tenofovir/emtricitabine
P6	M	Unknown- Hispanic/ Latino	Potentially Early	76	655,708	424	13	2,477	76	Efavirenz/Emtricitabine/ Tenogovir
P7	M	Black	Chronic	348	104,390	573	31	703	38	Elvitegravir/Cobicistat/Tenofovir/ Emtricitabine
P8	M	Black	Chronic	75	98,462	240	20	732	61	Rilpivirine/tenofovir/emtricitabine
P9	M	White	Chronic	35	170,733	152	15	495	49	Elvitegravir/ Cobicistat/ Emtricitabine/ Tenofovir DF
P10	M / Transgender F	Unknown- Hispanic/ Latino	Chronic	914	127,380	332	22	1,087	72	Rilpivirine/tenofovir/emtricitabine
P11	M	White	Chronic	116	42,089	180	8	1,418	63	Efavirenz/Emtricitabine/ tenofovir
P12	M	White	Potentially Early	69	189,000	873	36	1,285	53	Darunavir/ Emtricitabine/ Tenofavoir/ Maraviroc/ Raltegravir/Ritonavir

Characteristics at the last study time point							
Latest ART regimen at last study point	Age at last study time point (year)	Duration of ART at last study time point (years)	Plasma viremia	CD4 count	CD4 %	CD8 count	CD8 %
Abacavir/Dolutegravir/ Lamivudine	56	10.2	<40	565	33	668	39
TAF/FTC/Dolutegravir	57	10.3	<40	844	42	523	26
Abacavir/Dolutegravir/ Lamivudine	46	10	<40	699	30	489	21
Elvitegravir/Cobicistat/Tenofovir DF/ Emtricitabine	37	5.1	<40	521	31	437	26
Abacavir/Dolutegravir/ Lamivudine	61	5.2	<40	534	39	274	20
Elvitegravir/Cobicistat/TAF/ Emtricitabine	32	5.1	<40	557	35	366	23
Elvitegravir/Cobicistat/TAF/ Emtricitabine	28	5.4	<40	1085	39	974	35
Rilpivirine/tenofovir/emtricitabine	27	5.1	<40	986	49	684	34
Bictegravir/ tenofovir AF/ emtricitabine	38	4.8	<40	460	30	582	38
Bictegravir/ tenofovir AF/ emtricitabine	30	4.2	<40	965	42	1126	49
Rilpivirine/ Tenofovir/ emtricitabine	69	5	<40	260	22	485	41
Abacavir/lamibudine/atazanavir/dolutegravir/maraviroc	53	5.5	<40	1439	64	517	23

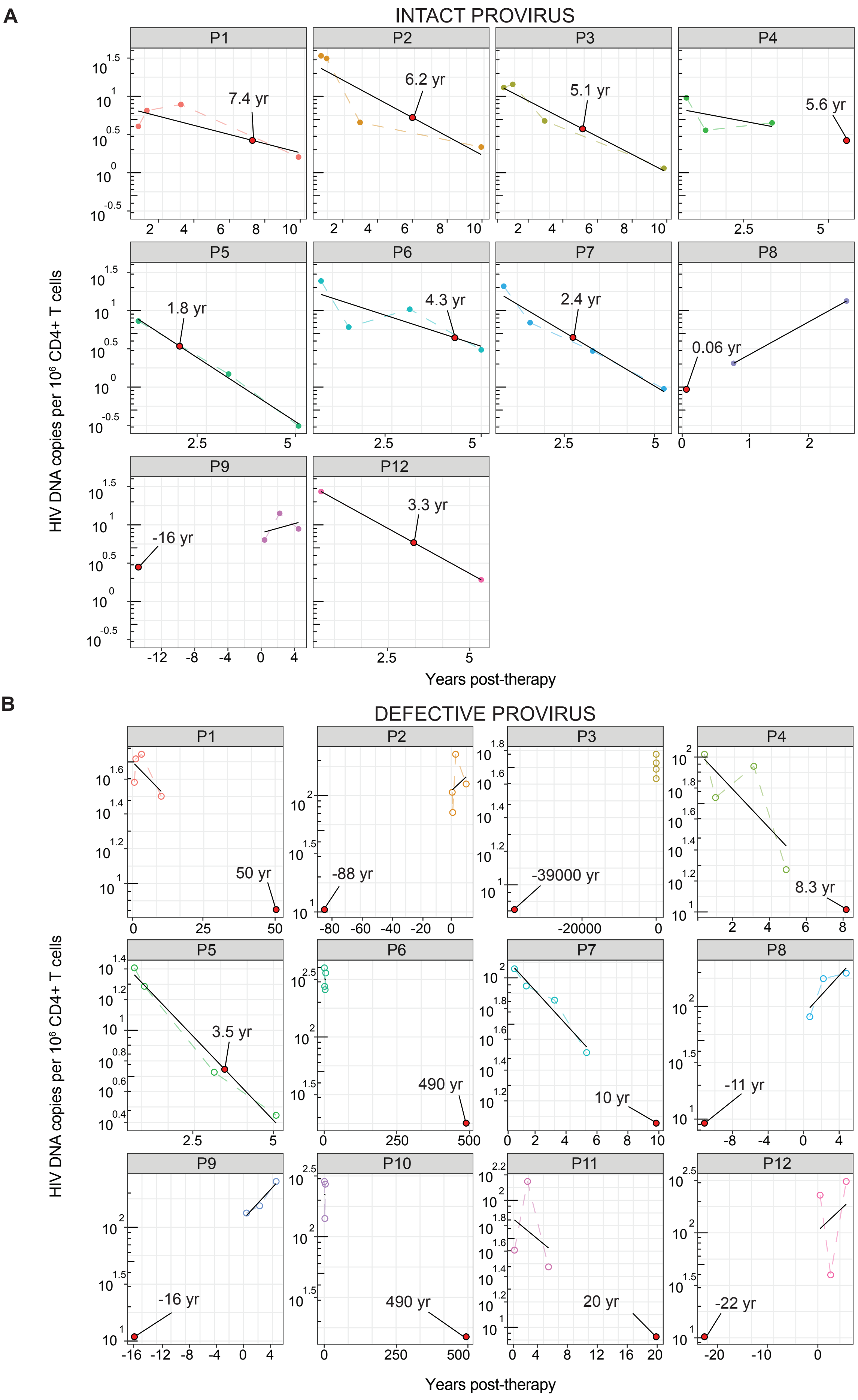

A
INTACT PROVIRUS
P1
P2
P3
P4
P5
P6
P7
P8
P9
P12
7.4 yr
6.2 yr
5.1 yr
5.6 yr
1.8 yr
4.3 yr
2.4 yr
0.06 yr
-16 yr
3.3 yr
HIV DNA copies per 10^6 CD4+ T cells
Years post-therapy
B
DEFECTIVE PROVIRUS
P1
P2
P3
P4
P5
P6
P7
P8
P9
P10
P11
P12
50 yr
-88 yr
-39000 yr
8.3 yr
3.5 yr
490 yr
10 yr
-11 yr
-16 yr
490 yr
20 yr
-22 yr
HIV DNA copies per 10^6 CD4+ T cells
Years post-therapy

Fig. S1. Half-life of the reservoir in each individual participant. The half-life of HIV proviral reservoir was calculated for each individual participant, for both (A) intact and (B) defective proviruses. Half-life is indicated by labeled red dot on each graph. The exponential decay half-life was calculated by using log10 IUPM values and a linear regression model.

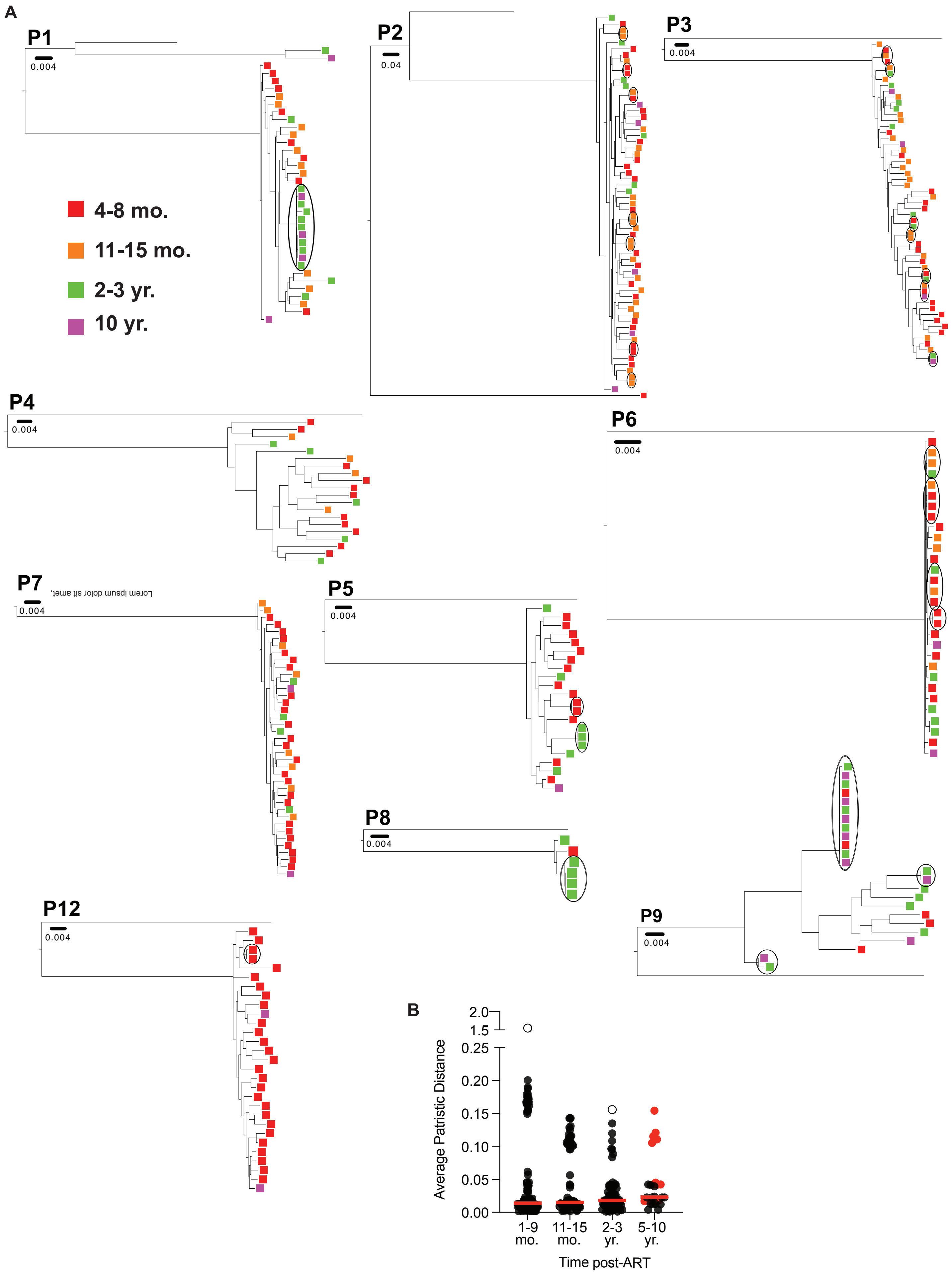
A
P1
0.004
4-8 mo.
11-15 mo.
2-3 yr.
10 yr.
P2
0.04
P3
0.004
P4
0.004
P6
0.004
P7
Lorem ipsum dolor sit amet.
0.004
P5
0.004
P8
0.004
P12
0.004
P9
0.004
B
Average Patristic Distance
2.0
1.5
0.25
0.20
0.15
0.10
0.05
0.00
1-9 mo.
11-15 mo.
2-3 yr.
5-10 yr.
Time post-ART

Fig. S2. Diversity of the reservoir does not change over the course of long-term ART treatment. (A) Phylogenic tree of total HIV sequence of intact proviruses found in all participants (N=10). Branch lengths are proportion to a genetic distance (scale bar indicated below each Sample ID). Clones are outlined in black. Color of squares represents when the sequence was detected in the reservoir, as indicated by the key on the left. (B) Graph showing average patristic distance between each intact provirus and the rest of the population from the same time point of the same individual. Each dot represents one intact provirus found in the reservoir. Unfilled dots represent outliers that were excluded from analysis. Data derived from participants sampled at 10 years post-ART are highlighted in red. Horizontal red line indicates the mean patristic distance at each time point. Grubb's test was used to calculate outliers, and A Kruskal-Wallis test with subsequent Dunn's multiple comparisons was used to analyze data where appropriate.

Table S2. Definition of defective categories

**Defective Category**	**Characteristics of category**
Missing Internal Genes	Truncated provirus, with large internal deletions spanning one or more coding genes
Non-coding	Full-length or truncated provirus, containing at least part of all genes, and early stop codons in one or more coding gene.
MSD Mutation	Full-length, in-frame, but lacking the 5' donor splice site 1 (D1)
Inversions or Duplications	Inversions or duplications detected in any genes or genomic sequence
Uncategorized	Unannotated defective due to missing either 3' LTR of 5' PS region where NFL primers should be binding

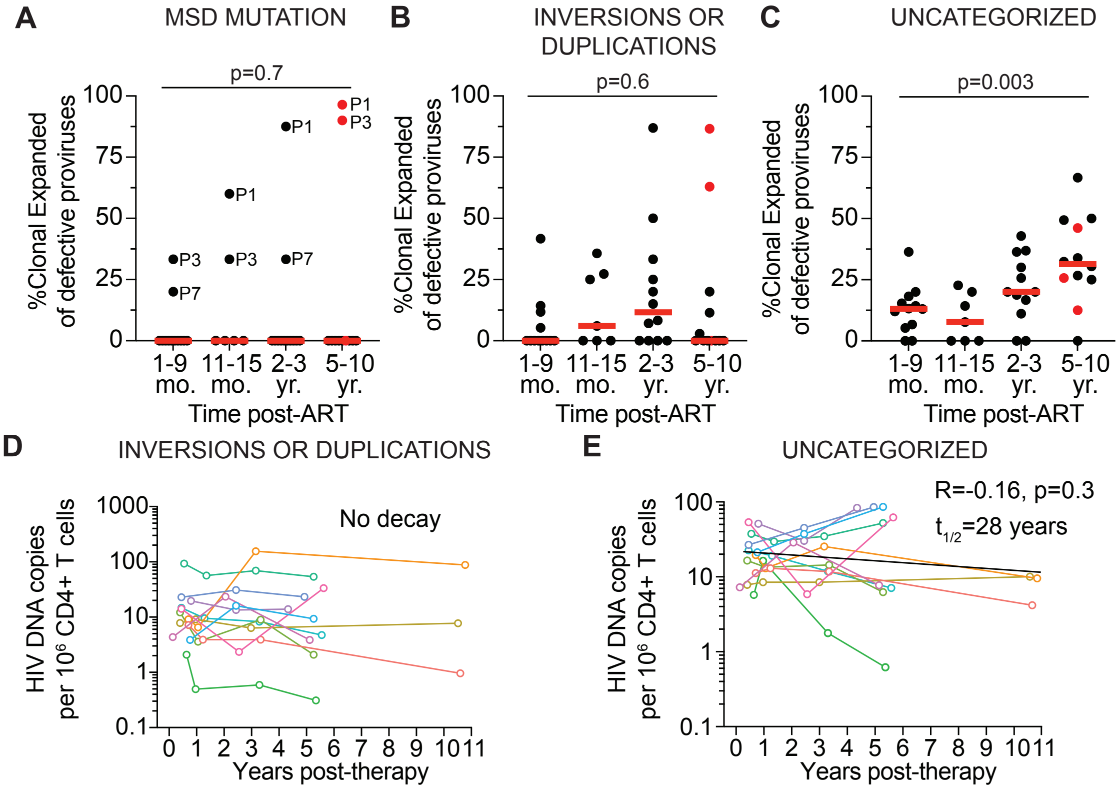

Fig. S3. Longitudinal analysis of different categories of defective proviral reservoirs. Frequency of clonally expanded defective proviruses per individual for defective categorized as (A) MSD mutation (Sample identifiers added for the few samples with clonally expanded defectives categorized as MSD mutation), (B) Inversion or duplications, or (C) Uncategorized. Participants sampled at 10 years post-ART are highlighted in red. Red horizontal line indicated median frequency. Graph showing the number of defective HIV-1 proviruses per million CD4+ T cells detected over time, when defectives are categorized as (D) Inversions of duplications (No decay, no half-life calculated) or (E) Uncategorized (Half-life = 28 years, R=-0.16, p=0.3). Each colored line represents one individual participant. A Kruskal-Wallis test with subsequent Dunn's multiple comparisons was used to analyze data where appropriate. The exponential decay half-life was calculated by using log10 IUPM values and a linear regression model.
